# Associations of Plasma Homocysteine Reflecting Vitamin B_12_ and Folate Status with Fatigue-Related Outcomes in Healthy Adults

**DOI:** 10.3390/nu18060941

**Published:** 2026-03-17

**Authors:** Hiroaki Kanouchi, Ayaka Yamamoto, Akiko Kuwabara, Shigeo Takenaka, Eiji Nishikubo, Yukihiro Nomura, Takehiro Naruto, Kyosuke Watanabe, Kei Mizuno, Yasuyoshi Watanabe

**Affiliations:** 1 Department of Nutrition, Graduate School of Human Life and Ecology, Osaka Metropolitan University, Osaka 536-8525, Japan; 2 Alinamin Pharmaceutical Co., Ltd., Tokyo 100-0005, Japan; 3 Kobe University Graduate School of Science, Technology and Innovation, Osaka 530-0011, Japan; 4 Center for Health Science Innovation, Osaka Metropolitan University, Osaka 558-8585, Japan

**Keywords:** homocysteine, fatigue, motivation, vitamin B_12_, folate, oxidative stress, cross−sectional study

## Abstract

**Background/Objectives**: Fatigue and reduced motivation impair daily functioning and quality of life. Homocysteine (Hcy) has been implicated in oxidative stress and one–carbon metabolism, but its relationship with fatigue–related outcomes remains unclear. We explored associations between plasma Hcy and fatigue-related measures in healthy adults. **Methods**: We analyzed cross–sectional data from 602 community–dwelling adults. Plasma Hcy concentrations were categorized into sex−specific tertiles. Fatigue and motivation were assessed using the Chalder Fatigue Scale and a visual analog scale (VAS). Sex−stratified multivariable linear models adjusted for lifestyle and biochemical covariates were used to examine associations. Sensitivity analyses additionally modeled Hcy as a continuous variable. **Results**: Higher Hcy tertiles were associated with lower serum folate and vitamin B_12_ concentrations in both sexes (*p* < 0.001). In men, the lowest Hcy tertile was associated with lower Chalder physical fatigue scores, whereas in women the highest Hcy tertile was associated with lower VAS motivation scores in multivariable analyses. Pairwise contrasts indicated higher physical fatigue in men in the highest tertile compared with the lowest (T3–T1: 1.55; 95% CI 0.24–2.86; *p* = 0.022) and lower motivation in women (T3–T1: −5.62; 95% CI −10.65 to −0.59; *p* = 0.029). However, no significant associations were observed when Hcy was modeled as a continuous variable. **Conclusions**: In this exploratory cross−sectional analysis, plasma Hcy showed associations with fatigue−related outcomes in sex−stratified analyses. These findings should be interpreted cautiously and considered hypothesis–generating. Longitudinal and mechanistic studies are needed to clarify potential causal relationships.

## 1. Introduction

Fatigue is a prevalent and debilitating symptom that poses a significant public health burden with wide–ranging socioeconomic implications. Chronic fatigue has been linked to reduced workplace productivity, increased absenteeism, and elevated healthcare utilization, collectively contributing to substantial economic costs worldwide [1,2]. In occupational settings, persistent fatigue can impair cognitive functioning, attention, and decision−making, thereby increasing the likelihood of errors and work–related accidents [3,4]. Moreover, fatigue–related declines in motivation may reduce engagement in health–promoting behaviors [5]. Identifying biological correlates or biomarkers of fatigue and motivation could offer critical insights for preventive strategies, early risk detection, and the development of targeted interventions, particularly in occupational and public health contexts.

Homocysteine (Hcy) is a sulfur−containing amino acid formed during methionine metabolism. Elevated plasma Hcy levels–referred to as hyperhomocysteinemia–have been associated with oxidative stress and impaired one-carbon metabolism [6,7,8], which may contribute to cardiovascular diseases, cognitive decline, and depression [9,10,11,12]. Circulating Hcy concentrations are influenced by genetic variants, renal function, and micronutrient status, particularly folate, vitamin B_12_ (VB_12_), and vitamin B_6_, which act as essential cofactors in Hcy metabolism [7,13]. Given its central role in one–carbon metabolism, methylation reactions, and monoamine neurotransmitter synthesis, Hcy may influence central nervous system function and contribute to fatigue-related phenotypes [12,14]. For example, Hcy accumulation can reduce the availability of S-adenosylmethionine (SAM), a universal methyl donor required for the synthesis of dopamine and serotonin, thereby impairing neuromodulation and neuroplasticity [15,16,17].

These neurochemical pathways are intimately involved in motivation [18,19]. Motivational decline and fatigue are increasingly recognized as related constructs within central fatigue syndromes, sharing common neurobiological substrates such as dopaminergic dysfunction and impaired prefrontal cortical activity [20,21]. Therefore, it is biologically plausible that elevated Hcy may not only contribute to diminishing motivational capacity but also fatigue. In addition, because Hcy reflects the integrated status of several B vitamins involved in one-carbon metabolism, Hcy may serve as a practical metabolic indicator for exploring fatigue-related outcomes in population-based studies.

Although previous studies have linked elevated Hcy levels to depression and cognitive impairment [11,12], little is known about its role in fatigue and motivational outcomes. Therefore, the present study aimed to examine the associations between circulating Hcy and multiple fatigue-related measures in community-dwelling adults. Because the biological mechanisms linking Hcy to fatigue are not fully established, these analyses were considered exploratory. Multivariable models were used to examine the associations between plasma Hcy and fatigue–related outcomes while adjusting for relevant lifestyle and biochemical covariates.

## 2. Materials and Methods

### 2.1. Study Design and Participants

This study used data from RIKEN Compass to Healthy Life Research Complex Program in Kobe, Japan, from April 2018 to March 2020. Participants were recruited through advertisement posters and the program website. Eligible individuals were healthy adults aged ≥18 years residing in Kobe and Osaka areas in Japan. A total of 2618 individuals visited the Center for Health Science Innovation (CHSI), Osaka Metropolitan University, where they underwent functional measurements and blood sampling and completed questionnaires between April 2018 and March 2020 (men: 783 [ca. 30%], women: 1835 [ca. 70%]; mean age: 44 years).

### 2.2. Exclusion Criteria

**Figure 1:**
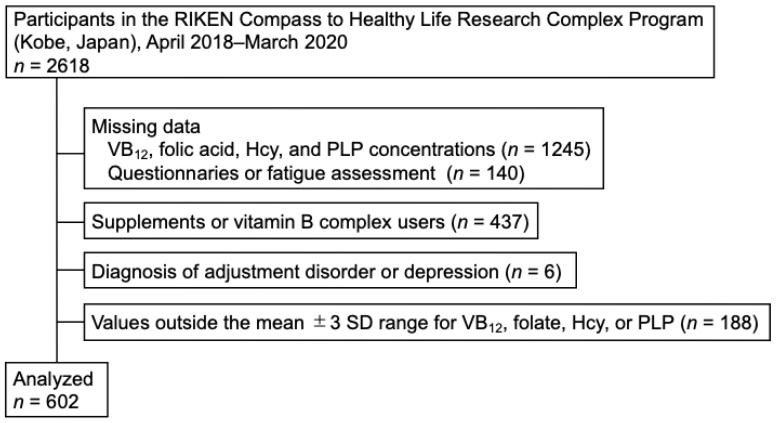
Flowchart for inclusion of study participants.

Participants were excluded if they had missing data for VB_12_, folate, Hcy, or co-enzyme form of vitamin B_6_, pyridoxal phosphate (PLP) concentrations (*n* = 1245); missing information on supplement use, daily medication, dietary variety, or fatigue assessments (*n* = 140); reported use of supplements or vitamin B complex (*n* = 437); had a diagnosis of adjustment disorder or depression (*n* = 6); or had biochemical values outside the mean ±3 SD for VB_12_, folate, Hcy, or PLP (*n* = 188). A total of 602 participants were included in the final analysis (men: 204; women: 398) (Figure 1). A large proportion of exclusions was due to the absence of stored blood samples available for Hcy measurement.

### 2.3. Blood Sampling

Blood samples were collected from the antecubital veins of the participants after at least 8 h of fasting.

### 2.4. Measurement of PLP

Serum PLP concentrations were measured using high-performance liquid chromatography (HPLC) following the protocol described by Itoh et al. [22]. Briefly, serum samples were deproteinized with trichloroacetic acid, derivatized with potassium cyanide under light–shielded conditions, and analyzed using an HPLC system (Shimadzu LC–20AD series, Shimadzu, Kyoto, Japan) equipped with a Chromolith column (UM8125/004, Merck, Darmstadt, Germany). The mobile phase consisted of 0.1 M citrate buffer (pH 3.5) containing 0.1% methanol, with a flow rate of 0.2 mL/min at 40 °C. PLP was detected by fluorescence (excitation at 418 nm, emission at 325 nm).

### 2.5. Measurement of Hcy

Plasma Hcy concentrations were determined by HPLC according to Mantjoro et al. [23], with minor modifications. Briefly, serum samples were added to N–acetyl–L–cysteine, which was used as an internal standard, deproteinized with trichloroacetic acid, conjugated with 4–Fluoro–7–sulfamoylbenzofurazan (DOJINDO, Kumamoto, Japan), and analyzed using an HPLC system (Shimadzu LC-20AD series) equipped with a Chromolith column (UM8125/004). The mobile phase consisted of 0.05 mol/L potassium dihydrogen phosphate (pH 1.9) combined with 30 mL/L acetonitrile, with a flow rate of 0.2 mL/min at 40 °C. Detection was performed by fluorescence (excitation at 385 nm, emission at 515 nm).

### 2.6. Measurement of Serum VB_12_ and Folate

Serum folate and VB_12_ concentrations were measured using commercially available kits: ACS–folic acid II kit (Bayer Medical, Osaka, Japan) and ACS–VB_12_ kit (Chiron Diagnostics, East Walpole, MA, USA), respectively. A fully automated ACS180 chemiluminescence analyzer (Bayer Diagnostics, Tarrytown, NY, USA) was used for the measurements. VB_12_ concentration was expressed as cyanocobalamin equivalents, and folic acid concentration was expressed as tetrahydrofolic acid equivalents (hereafter referred to as folate).

### 2.7. Fatigue Assessment

The Chalder Fatigue Scale is an 11–item self–reported questionnaire that evaluates both physical and mental fatigue [24,25]. Seven items represent physical fatigue (ChaPF) and 4 represent mental fatigue (ChaMF). Each item is scored 0–3; less than usual (0), no more than usual (1), more than usual (2) and much more than usual (3). Item ratings are summed to calculate the total score (ChaTF). The Visual Analog Scale (VAS) was used to measure the subjective intensity of both fatigue and motivation, using two separate 100 mm horizontal lines, where higher values represent greater fatigue or motivation, respectively. In addition, the CHSI Fatigue Scale, developed specifically for the Japanese population by Fukuda et al., was administered to assess multidimensional fatigue including physical, cognitive, and emotional domains [26]. This instrument has been validated for use in both healthy individuals and those with chronic fatigue syndrome. To evaluate psychological distress, we used the Kessler Psychological Distress Scale (K6), a six–item screening tool designed to measure nonspecific psychological distress experienced over the past 30 days [27]. Higher scores indicate greater psychological distress.

### 2.8. Questionnaires

Participants completed a digital questionnaire via a tablet, which collected information on body weight, height, medication use (type of medicine), supplement use (multi-vitamin, iron, coenzyme Q10, vitamin B, and vitamin C), and medical history (stroke, heart disease, anemia, hypertension, hyperuricemia, dyslipidemia, arrhythmia, kidney disease, gastroduodenal ulcer, depression, osteoporosis, liver disease, malignant tumor, and others).

Lifestyle factors included average working hours (≤7, >7–≤8 h, ≥8–≤9 h, >9–≤10 h, >10–≤11 h, or >11 h), sleep duration (≤5 h, >5–≤6 h, >6–≤7 h, >7–≤8 h, >8–≤9 h, or >9 h), and non-habitual exercise (<2 times/week). Overwork was defined as working more than 8 h per day, consistent with international labor standards (ILO Convention No.1) [28]. In accordance with World Health Organization (WHO) recommendations, lack of regular exercise was defined as engaging in physical activity <2 days/week [29]. Non–optimal sleep was defined as extreme sleep duration (<5 h or ≥9 h), based on previous epidemiological studies showing U–shaped associations between sleep duration and fatigue–related health outcomes [30,31,32]. Dietary habits were assessed using a dietary variety score, calculated based on the frequency of consumption of 10 food groups [33]. Responses of “eat almost every day” were assigned one point per group (maximum score: 10).

### 2.9. Ethics Statement

The study was approved by the Ethics Committee of RIKEN, a National Research and Development Agency in Japan (No. Kobe2 2017–04(11)) and the Ethics Committee of School of Human Life and Ecology, Osaka Metropolitan University (No. 23–25). The study was conducted in accordance with the principles of the Declaration of Helsinki. All participants provided written informed consent.

### 2.10. Statistical Analysis

Categorical variables are presented as numbers and percentages, and continuous variables as medians with interquartile ranges (25th–75th percentile). Descriptive statistics were computed using SPSS version 30 (IBM Corp., Armonk, NY, USA) and JMP version 14 (SAS Institute Inc., Cary, NC, USA). Trend tests for continuous variables were performed using the Jonckheere–Terpstra test in SPSS, and trend tests for categorical variables were conducted using the Cochran–Armitage test in JMP. Differences in continuous variables across Hcy tertiles were assessed using Steel’s test in JMP, with T1 as the reference group. Pearson correlation coefficients (r) and corresponding *p*–values for the heatmap were calculated in JMP. Multiple regression analyses were performed using JMP.

Plasma Hcy was categorized into tertiles (T1–T3). Because plasma Hcy concentrations are known to differ between men and women [7], all primary analyses were conducted separately by sex. We initially screened for associations between Hcy tertiles and multiple fatigue and motivation measures and identified significant relationships for ChaPF in men and VAS motivation in women. These measures were then selected for focused multivariable analyses. Multiple regression analysis was performed to assess the relationship between ChaPF and Hcy tertiles in men, and VAS motivation scores and Hcy tertiles in women, after adjusting for covariates (age, body mass index [BMI], non–optimal sleep, non–exercise habit, overwork, estimated glomerular filtration rate [eGFR], alanine aminotransferase [ALT], and dietary variety score). As a sensitivity analysis, plasma Hcy was also modeled as a continuous variable in multivariable regression models to examine potential linear associations with fatigue-related outcomes. Additionally, analyses including a sex–by–Hcy interaction term were performed using continuous Hcy in the multivariable models to evaluate potential sex differences.

Baseline characteristics were compared between participants included in the final analysis and those excluded from the analysis.

Estimated marginal means (EMMs), pairwise contrasts (T3–T1, T2–T1, T3–T2), and omnibus Wald χ^2^ tests for the figure in Section 3.4 were computed using Python (version 3.11) with the statsmodels (v0.14) and matplotlib libraries to ensure reproducibility and high-resolution visualization. Pairwise contrasts were expressed as adjusted mean differences (β) with 95% confidence intervals derived from the model variance–covariance matrix. An omnibus Wald χ^2^ test (2 *df*) assessed the overall effect. The figure in Section 3.4 visualizes adjusted pairwise contrasts among Hcy tertiles for ChaPF in men and VAS motivation in women. Sex–stratified multivariable linear models were fitted with indicator variables for Hcy tertiles (reference = T1) and adjusted for eGFR, non–optimal sleep, overwork, no exercise habit, age, BMI category, and dietary variety score. EMMs were computed at the sample mean of continuous covariates and observed proportions of categorical covariates.

To further explore the relationships among B–vitamin status, Hcy, lifestyle factors, and fatigue–related outcomes, exploratory path models were constructed separately for men and women. The models examined potential pathways linking folate, VB_12_, Hcy tertiles, lifestyle factors, and fatigue–related outcomes while accounting for relevant covariates. Path analyses were performed using SPSS Amos (IBM Corp.) as exploratory analyses.

## 3. Results

Baseline characteristics were compared between included and excluded participants (Supplementary Table S1). In men, age, eGFR, overwork, BAP, d–ROMs/BAP ratio, and VAS motivation differed between the two groups. In women, ALT, d–ROMs, d–ROMs/BAP ratio, non–habitual exercise, and VAS depression also differed between the two groups. However, ChaPF in men and VAS motivation in women—the outcomes examined in the present analyses—did not differ significantly between the included and excluded participants.

### 3.1. Biochemical Profiles, Lifestyle Characteristics and Fatigue Measures Among Hcy Tertiles

In men (*n* = 204), Hcy tertiles were defined as T1 < 14.6 μmol/L, T2 ≥ 14.6 to <17.6 μmol/L, and T3 ≥ 17.6 μmol/L (*n* = 68 each) (Table 1). In women (*n* = 398), Hcy tertiles were defined as T1 < 11.5 μmol/L, T2 ≥ 11.5 to <13.9 μmol/L, and T3 ≥ 13.9 μmol/L (Table 2). In women, eGFR was significantly lower in T2–T3 than in T1 (*p* < 0.001), whereas no significant trend was observed in men. Serum VB_12_ and folate decreased progressively across tertiles in both men and women (all *p* < 0.001). Median VB_12_ declined from 443 pg/mL in T1 to 365 pg/mL in T3 among men, and from 500 pg/mL to 413 pg/mL among women. Folate decreased from 9.85 ng/mL to 7.25 ng/mL in men and from 11.2 ng/mL to 9.0 ng/mL in women across tertiles. Oxidative stress markers, d–ROMs/BAP ratio, did not differ significantly across Hcy tertiles in either men or women (all *p* > 0.05).

In men, only ChaPF exhibited a positive trend across Hcy tertiles (*p* = 0.046) (Table 3). In women, VAS motivation exhibited a negative trend across Hcy tertiles (*p* = 0.024) and was significantly lower in T3 than in T1 (*p* = 0.024) (Table 4). A high score on the VAS motivation means good motivation. Therefore, subsequent analyses focused on ChaPF in men and VAS motivation in women.

The proportions of overwork (≥8 h/day), non-optimal sleep (<5 h or ≥9 h), and non-habitual exercise (<2 times/week) did not differ materially across Hcy tertiles in either sex (all *p* > 0.26 in men; all *p* > 0.58 in women).

### 3.2. The Relation Between Chalder Fatigue Scale Scores and VAS Motivation and Each Clinical and Lifestyle Factor

**Figure 2:**
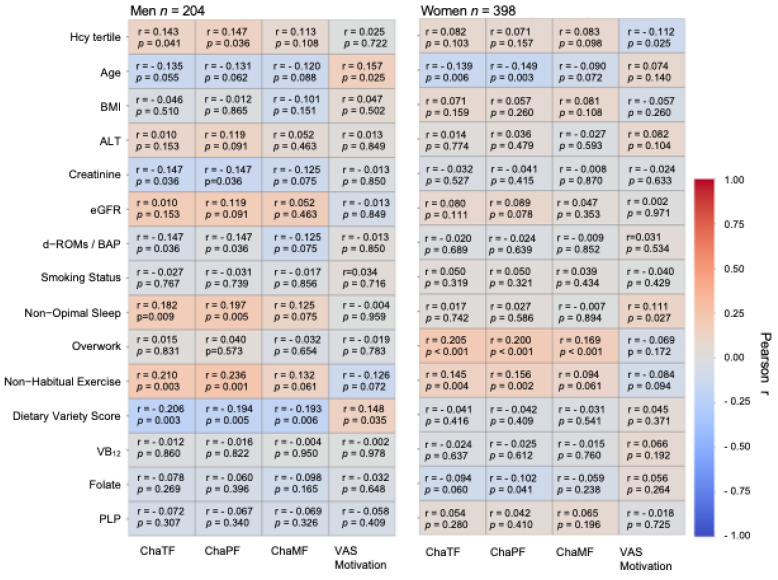
Pearson correlation heatmaps between fatigue and motivation scores and clinical/lifestyle variables in men (*n* = 204) and women (*n* = 398). Color scale represents correlation coefficients (r), with corresponding *p*-values annotated. Positive correlations are shown in red, negative correlations in blue. Hcy, homocysteine; BMI, body mass index; ALT, alanine transaminase; eGFR, estimated glomerular filtration rate; d-ROMs, diacron-reactive oxygen metabolites test; BAP, biological antioxidant potential; VB_12_, vitamin B_12_; PLP, pyridoxal phosphate.

Figure 2 presents heatmaps illustrating the associations of Chalder Fatigue Scale scores and VAS motivation with clinical and lifestyle factors. Pearson correlation analysis indicated that ChaPF in men were positively associated with Hcy tertile (r = 0.14, *p* = 0.04), eGFR (r = 0.187, *p* = 0.01), non-optimal sleep (r = 0.20, *p* = 0.01), and non-habitual exercise (r = 0.24, *p* = 0.01), whereas dietary variety score was negatively associated with Hcy tertile (r = −0.19, *p* = 0.01). In women, VAS motivation was significantly associated with Hcy tertile (r = −0.11, *p* = 0.03) and also showed moderate inverse correlations with Chalder Fatigue Scale scores, including ChaTF (r = −0.269), ChaPF (r = −0.240), and ChaMF (r = −0.245), all *p* < 0.001.

**Figure 3:**
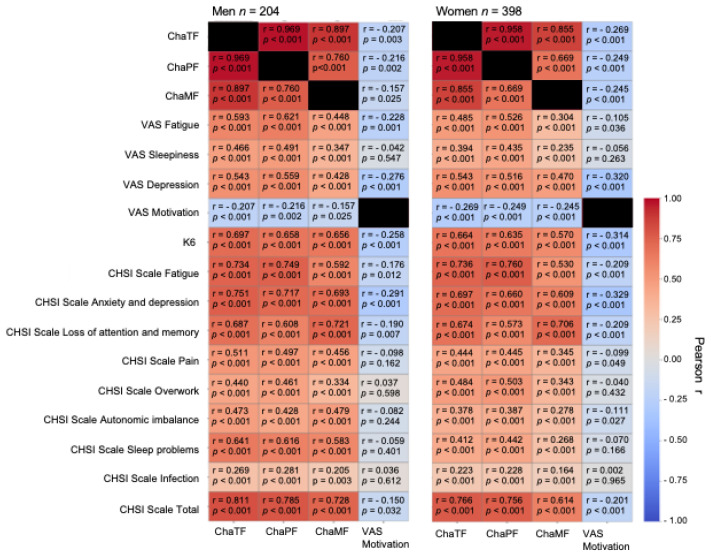
Pearson correlation heatmaps illustrating interrelationships among fatigue and motivation measures in men (*n* = 204) and women (*n* = 398). Color scale represents correlation coefficients (r), with corresponding *p*-values annotated. Positive correlations are shown in red, negative correlations in blue. Hcy, homocysteine; VAS, visual analog scale; K6, Kessler psychological distress scale 6; ChaTF, Chalder total fatigue; ChaPF, Chalder physical fatigue; ChaMF, Chalder mental fatigue; CHSI, Center for Health Science Innovation.

Figure 3 shows strong positive correlations among fatigue-related scales in both sexes (all r > 0.50, *p* < 0.001), including Chalder physical fatigue, Chalder mental fatigue, and CHSI total score. VAS motivation was inversely correlated with fatigue measures, but effect sizes were modest (r = −0.15 to −0.20).

### 3.3. Multivariable Models

Sex-stratified multivariable linear models were adjusted for eGFR, non-optimal sleep, overwork, non-habitual exercise, age, BMI category, and dietary variety score. In men, multivariable linear regression analysis showed that belonging to the lowest Hcy T1 was independently associated with lower ChaPF (β = −0.748, 95% CI −1.467 to −0.029, *p* = 0.042), whereas the Hcy T2 and T3 were not significantly associated with ChaPF (Table 5). Dietary variety was inversely associated with ChaPF. In women, multivariable linear regression analysis demonstrated that belonging to the highest Hcy T3 was independently associated with VAS motivation scores (β = −3.274, 95% CI −6.165 to −0.384, *p* = 0.027), while no significant associations were observed for the tertiles 1 or tertile 2 (Table 6). Non-optimal sleep was positively associated with VAS motivation. When Hcy was modeled as a continuous variable in the multivariable regression model, no significant association was observed with ChaPF in men (β = 0.68, 95% CI −0.18 to 1.53, *p* = 0.12). Similarly, continuous Hcy levels were not significantly associated with VAS motivation in women (β = −6.62, 95% CI −14.40 to 1.17, *p* = 0.096). In additional analyses including a sex-by-Hcy interaction term using continuous Hcy, no significant interaction between sex and plasma Hcy concentration was observed for ChaPF (*p* = 0.281) or VAS motivation (*p* = 0.511).

### 3.4. Pairwise Contrasts Based on Estimated Marginal Means

**Figure 4:**
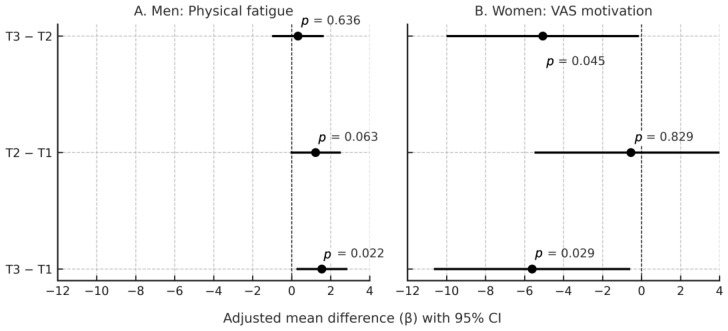
Adjusted pairwise contrasts of Hcy tertiles for fatigue and motivation outcomes (sex–separated panels). Forest plot displays adjusted mean differences (β) with 95% confidence intervals for pairwise contrasts among Hcy tertiles (T1–T3): T3–T1, T2–T1, T3–T2. Differences were derived from EMMs obtained from multivariable models. Models were adjusted for eGFR (continuous), non–optimal sleep (categorical: 0 = normal, 1 = <5 h or ≥9 h), overwork (categorical: 0 = <8 h, 1 = ≥8 h), non-habitual exercise (categorical: 0 = ≥ 2 times/week, 1 = <2 times/week), age (continuous), BMI category (<18.5, 18.5–24.9, ≥25.0; reference = 18.5–24.9), and dietary variety score (continuous). Outcomes: Chalder Physical Fatigue (men, *n* = 204) and VAS motivation (women, *n* = 398). Positive values indicate higher scores in the first tertile listed; negative values indicate lower scores.

To clarify the relationships among Hcy tertiles for Chalder Physical Fatigue and VAS motivation, EMMs and pairwise contrasts were derived from the multivariable regression models and visualized in Figure 4. These post hoc contrasts were conducted for descriptive purposes and were not directly tested in the original regression analyses presented in Table 5 and Table 6. In men, adjusted EMMs for ChaPF were 5.83 (T1), 7.06 (T2), and 7.38 (T3). The contrast T3–T1 was +1.55 (95% CI 0.24–2.86, *p* = 0.022), indicating significantly higher ChaPF in the highest Hcy tertile. The contrasts T2–T1 (+1.23, *p* = 0.063) and T3–T2 (+0.32, *p* = 0.636) were not significant. The omnibus test for Hcy tertiles was borderline (Wald χ^2^ = 6.11, *p* = 0.047). In women, adjusted EMMs for VAS motivation were 53.76 (T1), 53.21 (T2), and 48.14 (T3). The contrast T3–T1 was −5.62 (95% CI −10.65 to −0.59, *p* = 0.029), indicating significantly lower motivation in T3. The contrast T3–T2 was also significantly low −5.07 (95% CI −10.00 to −0.14, *p* = 0.045). On the other hand, the contrasts T2–T1 ( −0.55, *p* = 0.829) was not significant. The omnibus test approached significance (Wald χ^2^ = 5.89, *p* = 0.053). Note: The pairwise contrast results shown in Figure 4 are based on EMMs and differ from the multivariable regression estimates presented in Table 5 and Table 6 because they arise from different analytic frameworks.

### 3.5. Additional Analyses with Folate and VB_12_

To examine whether associations between Hcy tertiles and fatigue-related outcomes were attributable to underlying vitamin status, serum folate and VB_12_ concentrations were evaluated as predictors using the same multivariable models. In men, neither folate tertiles nor VB_12_ tertiles were significantly associated with ChaPF (folate: T1 [β = −0.035, 95% CI −0.776 to 0.706, *p* = 0.926], T2 [β = −0.170, 95% CI −0.910 to 0.569, *p* = 0.650], T3 [β = 0.205, 95% CI −0.550 to 0.960, *p* = 0.592]; VB_12_: T1 [β = −0.366, 95% CI −1.088 to 0.355, *p* = 0.318], T2 [β = 0.128, 95% CI −0.597 to 0.854, *p* = 0.728], T3 [β = 0.238, 95% CI −0.487 to 0.962, *p* = 0.518]). In women, neither folate tertiles nor VB_12_ tertiles were significantly associated with VAS motivation (folate: T1 [β = −0.189, 95% CI −3.209 to 2.831, *p* = 0.902], T2 [β = 0.937, 95% CI −1.934 to 3.808, *p* = 0.521], T3 [β = −0.748, 95% CI −3.764 to 2.267, *p* = 0.626]; VB_12_: T1 [β = −0.439, 95% CI −3.375 to 2.497, *p* = 0.769], T2 [β = −0.863, 95% CI −3.725 to 1.999, *p* = 0.554], T3 [β = 1.303, 95% CI −1.596 to 4.201, *p* = 0.377]). These findings suggest that the observed associations between Hcy and fatigue-related outcomes are unlikely to be explained solely by folate or VB_12_ status.

### 3.6. Exploratory Path Models

**Figure 5:**
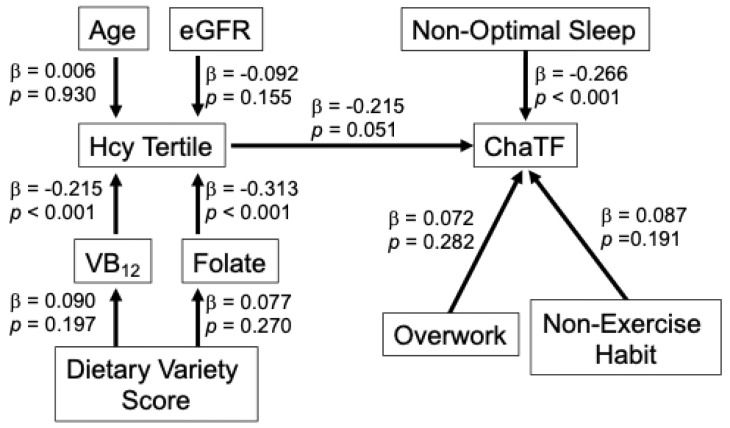
Exploratory path model showing the relationships among B vitamins, Hcy, lifestyle factors, and ChaPF in men. The model illustrates the associations among folate, VB_12_, Hcy tertiles, lifestyle factors (non-optimal sleep, overwork, and non-exercise habit), and ChaPF, while accounting for age, renal function (eGFR), and dietary variety score. Standardized path coefficients (β) and *p* values are shown.

**Figure 6:**
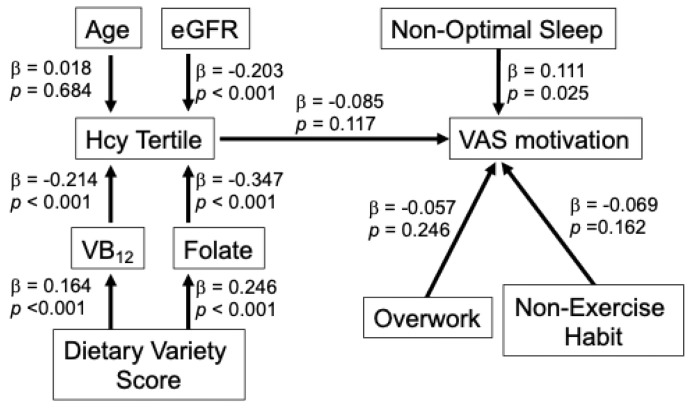
Exploratory path model showing the relationships among B vitamins, Hcy, lifestyle factors, and VAS motivation in women. The model illustrates the associations among folate, VB_12_, Hcy tertiles, lifestyle factors (non–optimal sleep, overwork, and non-exercise habit), and VAS motivation, while accounting for age, renal function (eGFR), and dietary variety score. Standardized path coefficients (β) and *p* values are shown.

To further examine the relationships among B–vitamin status, Hcy, lifestyle factors, and fatigue-related outcomes, and fatigue–related outcomes, path models were constructed separately for men (Figure 5) and women (Figure 6). The models indicated that folate and VB_12_ were significantly associated with Hcy tertiles, whereas their direct associations with fatigue-related outcomes were not significant. In men, higher Hcy levels were modestly associated with ChaPF, while in women a similar pathway structure was observed with a weaker association between Hcy and VAS motivation. These findings suggest that B-vitamin status may influence fatigue–related outcomes primarily through its relationship with Hcy.

## 4. Discussion

Elevated Hcy was associated with ChaPF in men and VAS motivation in women in the multivariable analyses after adjustment for sleep, work hours, exercise, BMI, dietary variety, and renal function. These associations should be interpreted as exploratory.

Fatigue is increasingly recognized as a multidimensional construct that includes physical, cognitive, and emotional components. The Chalder Fatigue Scale primarily captures physical and mental fatigue [24], whereas the CHSI scale assesses broader symptom domains, including cognitive and autonomic complaints [26]. In contrast, the VAS motivation score may be sensitive to short–term fluctuations in motivation. Examining multiple scales, therefore, allowed us to explore whether Hcy may be differentially associated with specific dimensions of fatigue-related experiences. Fatigue primarily reflects a subjective perception of physical or mental exhaustion, whereas motivational decline reflects reduced drive to initiate or sustain goal-directed behavior. Although related, these constructs represent distinct aspects of central fatigue syndromes [34].

Hcy is a key intermediate in one-carbon metabolism, requiring folate and VB_12_ for remethylation and vitamin B_6_ for transsulfuration [7]. In both sexes, higher Hcy tertiles corresponded to lower folate and VB_12_, consistent with impaired clearance when these cofactors are relatively low. However, folate and VB_12_ were not independently associated with fatigue or motivation, suggesting that Hcy may reflect a broader metabolic state beyond micronutrient status. Mechanistically, elevated Hcy has been proposed to influence methylation processes through reductions in SAM, a universal methyl donor involved in monoamine synthesis [12,15,16,17]. Such mechanisms could potentially influence dopaminergic and serotonergic signaling implicated in motivation and fatigue [18,19,20,21], although these pathways were not directly assessed in the present study.

The observed sex–specific patterns may reflect differences in Hcy metabolism and neural regulation between men and women, although these interpretations remain speculative. Plasma Hcy concentrations are generally lower in women than in men, partly due to the influence of sex hormones such as estrogen on Hcy metabolism [9]. In addition, sex hormones can modulate central dopaminergic systems; for example, estrogen has been reported to alter dopamine transporter and receptor expression in the brain [35]. Additionally, structural and functional sex differences in the prefrontal cortex and striatum may also contribute to distinct vulnerabilities to metabolic stressors [36,37,38,39].

Although Hcy has been implicated in promoting oxidative stress and excitotoxicity in previous studies [6,40], our findings did not show significant differences in the d-ROMs/BAP ratio across Hcy tertiles. Epidemiological studies have reported positive associations between Hcy and thiobarbituric acid reactive substances (TBARS), a lipid peroxidation marker, in pathological conditions such as central retinal vein occlusion and schizophrenia [41,42]. However, no studies have examined the relationship between Hcy and d-ROMs/BAP ratio, and previous reports indicate that TBARS and d-ROMs may not correlate consistently [43]. Therefore, the absence of association in our data does not exclude a potential link between Hcy and oxidative stress.

From a clinical perspective, maintaining adequate B-vitamin status to support Hcy metabolism remains reasonable given the established links between Hcy and vascular and cognitive outcomes [9,10]. In general, plasma Hcy concentrations below 15 μmol/L are often considered within the normal range [9]; however, there is no universally accepted cutoff, and values may vary with population characteristics, assay methods, and clinical condition. For example, thresholds for defining hyperhomocysteinemia differ across studies and may be lower in neurological and cognitive outcomes [13]. In our study, the highest tertile in men (≥17.6 μmol/L) exceeded this threshold, while the highest tertile in women (≥13.9 μmol/L) approached it. These findings may suggest a possible association between Hcy levels and fatigue-related outcomes; however, the results should be interpreted cautiously given the exploratory nature of the analyses and the cross-sectional design. Hcy may reflect broader metabolic conditions that could be related to fatigue-related outcomes.

Consistent with this interpretation, folate and VB_12_ were strongly associated with lower Hcy concentrations [7,13], whereas their direct associations with fatigue-related outcomes were negligible, suggesting that B–vitamin status may influence fatigue primarily through its relationship with Hcy.

To further explore the relationships among B-vitamin status, Hcy, and fatigue–related outcomes, exploratory path models were constructed. These models indicated that folate and VB_12_ were significantly associated with Hcy tertiles, consistent with the known collinearity among these variables, whereas their direct associations with fatigue-related outcomes were not significant. This pattern suggests that B–vitamin status may influence fatigue-related outcomes primarily through its relationship with Hcy rather than through independent pathways. These findings should be interpreted cautiously.

Although these findings raise the possibility that nutritional strategies aimed at lowering Hcy may influence fatigue–related outcomes, causal relationships cannot be established from this cross-sectional study. Prospective and interventional studies are required to determine whether lowering Hcy can directly improve fatigue or motivational symptoms.

This study has several limitations that should be considered when interpreting the findings. First, the cross-sectional design precludes causal inference, and reverse causality cannot be excluded. Second, although Hcy tertiles were used to facilitate interpretation, Hcy did not show significant associations with fatigue or motivation when modeled as a continuous variable, suggesting possible threshold or non-linear effects. Therefore, tertile categorization was used to aid interpretation of potential non-linear relationships between Hcy and fatigue-related outcomes. Third, all fatigue and motivation measures were self-reported, which may not fully capture neurobiological correlates. Fourth, inflammatory processes have also been implicated in fatigue, with inflammatory cytokines such as IL-6 suggested as potential contributors to fatigue perception [43]. However, inflammatory biomarkers were not measured in the present study. Fifth, residual confounding due to unmeasured factors such as stress or sex hormones remains possible. Sixth, participants were recruited through advertisements for a preventive health program and may therefore represent a relatively health-conscious cohort, which may limit generalizability to the broader population. Finally, although sex–specific associations were identified, mechanistic biomarkers—such as SAM/SAH ratios, neurotransmitter metabolites, or neuroimaging indicators—were not assessed. Because these analyses were exploratory, the findings should be interpreted cautiously and considered hypothesis-generating. Longitudinal and mechanistic studies are needed to establish causality and clarify the biological basis of the sex differences observed [36].

## 5. Conclusions

In this community-based cohort, elevated Hcy was associated with ChaPF in men and VAS motivation in women, independent of lifestyle factors and measured B-vitamin status. These findings support a model in which Hcy reflects metabolic conditions both affected by stresses and micronutrient statuses. Further longitudinal and mechanistic studies incorporating endocrine measures, oxidative stress panels, and neurobiological markers are warranted. Future prospective and interventional studies are needed to determine whether reducing Hcy levels can influence fatigue-related outcomes.

## Figures and Tables

**Table 1:** Biochemical Characteristics of Participants in Men by Hcy Tertiles.

	Total	Hcy Tertiles (μmol/L)			
	*n* = 204	T1 (<14.6) *n* = 68	T2 (≥14.6, <17.6) *n* = 68	T3 (≥17.6) *n* = 68	*p* for Trend
Hcy (μmol/L)	16.1 (13.7–18.8)	13.0 (12.2–13.8)	16.1 (15.5–16.9) **	18.7 (18.1–20.2) **	<0.001
Age (years)	42 (34–51)	43.5 (35.5–50)	44 (34.3–52.8)	40.5 (3–51)	0.718
BMI (kg/m^2^)	22.4 (21.0–24.4)	22.4 (21.0–24.7)	23.2 (21.4–25.1)	22.0 (20.8–23.5)	0.234
ALT (U/L)	20 (15–28)	20.5 (15–29)	22 (16–32)	19 (15–25)	0.492
eGFR (mL/min/1.73 m^2^)	80.0 (71.6–91.1)	80.3 (72.0–92.1)	79.7 (71.2–87.6)	80.2 (71.6–93.0)	0.830
Creatinine (mg/dL)	0.86 (0.78–0.92)	0.86 (0.77–0.9)	0.87 (0.78–0.92)	0.86 (0.79–0.91)	0.874
d-ROMs (U.CARR)	321 (275–368)	317 (275–364)	328 (277–394)	320 (274–365)	0.749
BAP (μmol/L)	2415 (2229–2539)	2371 (2193–2532)	2356 (2224–2528)	2455 (2308–2566)	0.044
d-ROMs/BAP	0.13 (0.11–0.16)	0.13 (0.11–0.16)	0.14 (0.12–0.17)	0.13 (0.11–0.16)	0.558
VB_12_ (pg/mL)	404 (334–492)	443 (380–569)	394 (319–487) **	365 (314–429) **	<0.001
Folate (ng/mL)	8.7 (6.7–11.0)	9.85 (8.1–12.1)	9.50 (6.7–11.5)	7.25 (5.7–8.6) **	<0.001
PLP (nM)	50.8 (38.1–66.2)	56.2 (37.0–70.8)	49.8 (39.3–66.2)	48.6 (36.4–63.4)	0.251

Hcy, homocysteine; BMI, body mass index; ALT, alanine transaminase; eGFR, estimated glomerular filtration rate; d-ROMs, diacron-reactive oxygen metabolites test; BAP, biological antioxidant potential; VB_12_, vitamin B_12_; PLP, pyridoxal phosphate; ** *p* < 0.01 compared to T1.

**Table 2:** Biochemical Characteristics of Participants in Women by Hcy Tertiles.

	Total	Hcy Tertiles (μmol/L)			
	*n* = 398	T1 (<11.5) *n* = 132	T2 (≥11.5, <13.9) *n* = 133	T3 (≥13.9) *n* = 133	*p* for Trend
Hcy (μmol/L)	12.8 (10.8–14.8)	10.2 (9.37–10.8)	12.8 (12.3–13.3) **	16.1 (14.8–17.6) **	<0.001
Age (years)	45 (34–52)	43.4 (11.1–45)	45 (32–52.5)	47 (33–54)	0.078
BMI (kg/m^2^)	20.6 (19.1–22.5)	20.4 (19.0–22.9)	20.5 (19.0–22.3)	20.8 (19.3–22.3)	0.974
ALT (U/L)	12 (8–16)	12 (9–16)	13 (11–16)	12 (10–17)	0.999
eGFR (mL/min/1.73 m^2^)	83.1 (74.2–95.2)	87.3 (78.2–103)	81.3 (75.1–91.4) **	80.6 (69.4–92.1) **	<0.001
Creatinine (mg/dL)	0.62 (0.56–0.67)	0.59 (0.53–0.65)	0.63 (0.58–0.67) **	0.63 (0.7–0.58) **	<0.001
d-ROMs (U.CARR)	377 (333–419)	379 (338–420)	362 (326–417)	381 (335–419)	0.857
BAP (μmol/L)	2373 (2257–2504)	2343 (2224–2482)	2384 (2282–2517)	2385 (2274–2516)	0.104
d-ROMs/BAP	0.16 (0.14–0.18)	0.16 (0.14–0.18)	0.15 (0.13–0.18)	0.16 (0.14–0.18)	0.681
VB_12_ (pg/mL)	456 (356–556)	500 (413–599)	451 (351–538) **	413 (326–532) **	<0.001
Folate (ng/mL)	9.8 (7.9–12.7)	11.2 (9–14.2)	9.4 (8.05–12.4) **	9.0 (6.8–11.3) **	<0.001
PLP (nM)	41.1 (32.0–54.8)	41.4 (31.7–54.6)	40.8 (31.8–55.7)	41.0 (33.2–56.5)	0.811

Hcy, homocysteine; BMI, body mass index; ALT, alanine transaminase; eGFR, estimated glomerular filtration rate; d-ROMs, diacron-reactive oxygen metabolites test; BAP, biological antioxidant potential; VB_12_, vitamin B_12_; PLP, pyridoxal phosphate; ** *p* < 0.01 compared to T1.

**Table 3:** Lifestyle Habits and Fatigue Measures in Men by Hcy Tertiles.

	Total	Hcy Tertiles (μmol/L)			
	*n* = 204	T1 (<14.6) *n* = 68	T2 (≥14.6, <17.6) *n* = 68	T3 (≥17.6) *n* = 68	*p* for Trend
Overwork ^a^	140 (69)	49 (72)	45 (66)	46 (68)	0.579
Non-Optimal Sleep ^b^	28 (14)	8 (12)	11 (16)	9 (13)	0.803
Non-Habitual Exercise ^c^	182 (89)	59 (87)	60 (88)	63 (93)	0.269
Smoking Status Current	44 (22)	11 (16)	17 (25)	16 (24)	
Former	76 (37)	20 (29)	29 (43)	27 (40)	0.104
Never	84 (41)	37 (54)	22 (32)	25 (37)	
Dietary Variety Score	85.7 (71.4–100)	85.7 (71.4–100)	85.7 (71.4–100)	85.7 (71.4–100)	0.668
VAS Fatigue	30 (17–48)	25 (14–47.3)	30.5 (19.3–48.5)	31.5 (21.3–48.8)	0.058
VAS Sleepiness	32 (18.3–51.8)	29 (17–47.8)	36.5 (16–57.5)	34 (22.3–54.3)	0.108
VAS Depression	17.5 (0–35)	17.0 (0–33)	14.5 (0–43.8)	20.5 (7.25–37)	0.197
VAS Motivation	60 (48–76)	60 (48–78)	57.5 (48–74)	60 (50–77.8)	0.646
K6	1 (0–4)	1 (0–3)	1 (0–4)	2 (0–5)	0.119
ChaTF	10 (6–14)	9 (5.25–13)	10 (6–14)	11 (8–16)	0.053
ChaPF	6 (4–9)	6 (3–8)	6 (3–9)	7 (5–10)	0.046
ChaMF	4 (2–5)	4 (2–5)	4 (2–5)	4 (3–6)	0.130
CHSI Scale					
Total	3.8 (1.9–5.6)	3.8 (1.9–5.6)	3.4 (1.9–6.3)	3.8 (2.5–5.6)	0.432
Fatigue	3.5 (1.5–5.0)	3.0 (1.0–5.0)	3.0 (1.5–4.5)	3.5 (2.0–5.8)	0.167
Anxiety and depression	5.0 (2.0–6.0)	4.5 (2–6)	5 (3–6)	5 (3–7)	0.246
Loss of attention and memory	2.0 (1.0–4.0)	2.0 (1.0–4.0)	2.0 (1.0–4.0)	2.0 (1.0–3.8)	0.938
Pain	5.0 (2.0–8.0)	4 (2.0–7.0)	4 (2–7)	5 (3–9)	0.156
Overwork	0 (0–1.3)	0 (0–1.3)	0 (0–1.3)	0 (0–1.3)	0.986
Autonomic imbalance	3.3 (1.67–5.0)	3.3 (1.7–5.0)	3.3 (1.7–6.7)	3.3 (1.7–5.0)	0.994
Sleep problems	0 (0–2.5)	0 (0–2.5)	0 (0–2.5)	0 (0–0)	0.193
Infection	23 (15–34)	23 (14–33)	22 (14–37)	27 (18–35)	0.220

Hcy, homocysteine; VAS, visual analog scale; K6, Kessler psychological distress scale 6; ChaTF, Chalder total fatigue; ChaPF, Chalder physical fatigue; ChaMF, Chalder mental fatigue; CHSI, Center for Health Science Innovation. ^a^ ≥ 8 h, ^b^ < 5 h or ≥9 h, ^c^ < 2 days in a week.

**Table 4:** Lifestyle Habits and Fatigue Measures in Women by Hcy Tertiles.

	Total	Hcy Tertiles (μmol/L)			
	*n* = 398	T1 (<11.5) *n* = 132	T2 (≥11.5, <13.9) *n* = 133	T3 (≥13.9) *n* = 133	*p* for Trend
Overwork ^a^	110 (28)	36 (27)	36 (27)	38 (29)	0.813
Non-Optimal Sleep ^b^	39 (10)	12 (9)	13 (10)	14 (11)	0.694
Non-Habitual Exercise ^c^	351 (88)	118 (89)	117 (88)	116 (87)	0.583
Smoking Status Current	15 (4)	4 (3)	4 (3)	7 (5)	
Former	49 (12)	16 (12)	16 (12)	17 (13)	0.859
Never	334 (84)	112 (85)	113 (85)	109 (82)	
Dietary Variety Score	100 (75–100)	100 (75–100)	100 (88–100)	87.5 (75–100)	0.319
VAS Fatigue	38 (22–53)	38 (24–51)	35 (20–54)	38 (21–54)	0.908
VAS Sleepiness	34 (20–52)	37.5 (22–51)	32 (18–51)	35 (21–56)	0.660
VAS Depression	24 (9–43)	23 (9–41)	23 (7–41)	25 (11–48)	0.445
VAS Motivation	51 (41–67)	52 (45–69)	53 (43–70)	49 (36–60) *	0.024
K6	2 (0–6)	1 (0–4)	2 (0–6)	3 (0–7)	0.069
ChaTF	12 (8–16)	12 (8–15.8)	12 (8–16)	12 (9–17)	0.142
ChaPF	7 (5–11)	7 (5–10)	8 (5–11)	7 (5–12)	0.234
ChaMF	4 (3–6)	4 (3–6)	4 (3–6)	4 (3–6)	0.190
CHSI Scale					
Total	29.9 (19.6–41.7)	28.0 (18.8–36.6)	29.2 (18.7–40.1)	33.3 (20.9–46.4)	0.040
Fatigue	4.4 (2.5–7.5)	4.38 (2.5–6.9)	4.4 (2.5–6.9)	5.0 (2.8–8.8)	0.119
Anxiety and depression	4.5 (2.5–6.5)	3.5 (2.1–5.5)	4.0 (2.0–5.5)	4.5 (2.5–7.0)	0.044
Loss of attention and memory	5.0 (4.0–8.0)	5.0 (4.0–7.0)	5.0 (4.0–8.0)	6.0 (4.0–9.0)	0.036
Pain	3.0 (1.0–5.0)	3.0 (1.0–5.0)	3.0 (1–5)	3.0 (1.0–6.0)	0.942
Overwork	4.0 (2.0–7.0)	4.0 (2.0–6.0)	4.0 (2–7)	4.0 (2.0–8.0)	0.222
Autonomic imbalance	0.6 (0–1.3)	0 (0–1.3)	1.3 (0–2.5) **	1.3 (0–1.9)	0.058
Sleep problems	5.0 (1.7–6.7)	3.3 (1.7–6.7)	3.3 (1.7–6.7)	5.0 (3.3–8.3)	0.109
Infection	0 (0–2.5)	0 (0–2.5)	0 (0–2.5)	0 (0–2.5)	0.900

Hcy, homocysteine; VAS, visual analog scale; K6, Kessler psychological distress scale 6; ChaTF, Chalder total fatigue; ChaPF, Chalder physical fatigue; ChaMF, Chalder mental fatigue; CHSI, Center for Health Science Innovation. ^a^ ≥ 8 h, ^b^ < 5 h or ≥9 h, ^c^ < 2 days in a week. * *p* < 0.05 compared to T1; ** *p* < 0.01 compared to T1.

**Table 5:** Multiple Regression Analysis of Chalder Fatigue Scale Score and VAS motivation and Hcy Tertiles in Men.

	ChaTF		ChaPF		ChaMF		VAS Motivation	
	β [95% CI]	*p*	β [95% CI]	*p*	β [95% CI]	*p*	β [95% CI]	*p*
Hcy T1	−0.959 [−2.023, 0.105]	0.077	−0.748 [−1.467, −0.029]	0.042	−0.211 [−0.627, 0.205]	0.319	−0.664 [−5.281, 3.953]	0.777
Hcy T2	0.112 [−0.964, 1.188]	0.838	0.216 [−0.511, 0.944]	0.558	−0.104 [−0.526, 0.317]	0.626	−1.525 [−6.196, 3.147]	0.521
Hcy T3	0.847 [−0.233, 1.926]	0.124	0.532 [−0.198, 1.261]	0.153	0.315 [−0.107, 0.738]	0.143	2.189 [−2.497, 6.875]	0.358
Non-Optimal Sleep ^a^	1.595 [0.487, 2.703]	0.005	1.149 [0.400, 1.897]	0.003	0.446 [0.013, 0.880]	0.044	−0.455 [−5.263, 4.353]	0.852
Overwork ^b^	−0.258 [−1.085, 0.570]	0.539	−0.089 [−0.649, 0.470]	0.753	−0.169 [−0.492, 0.155]	0.306	0.012 [−3.579, 3.604]	0.995
Non-Exercise Habit ^c^	1.903 [0.660, 3.145]	0.003	1.428 [0.588, 2.268]	0.001	0.474 [−0.012, 0.960]	0.056	−5.121 [−10.51, 0.272]	0.063
Dietary Variety Score	−2.195 [−3.985, −0.406]	0.017	−1.421 [−2.630, −0.211]	0.022	−0.775 [−1.475, −0.074]	0.030	6.743 [−1.023, 14.510]	0.088

Hcy, homocysteine; ChaTF, Chalder total fatigue; ChaPF, Chalder physical fatigue; ChaMF, Chalder mental fatigue; VAS, visual analog scale. ^a^ ≥ 8 h, ^b^ < 5 h or ≥9 h. The model was adjusted with age, BMI, eGFR and ALT. ^c^ < 2 days in a week.

**Table 6:** Multiple Regression Analysis of Chalder Fatigue Scale Score and VAS motivation and Hcy Tertiles in Women.

	ChaTF		ChaPF		ChaMF		VAS Motivation	
	β [95% CI]	*p*	β [95% CI]	*p*	β [95% CI]	*p*	β [95% CI]	*p*
Hcy T1	−0.599 [−1.404, 0.206]	0.144	−0.420 [−0.981, 0.142]	0.142	−0.179 [−0.496, 0.138]	0.267	2.035 [−0.868, 4.937]	0.169
Hcy T2	−0.064 [−0.851, 0.724]	0.874	0.029 [−0.520, 0.579]	0.916	−0.093 [−0.403, 0.217]	0.555	1.240 [−1.601, 4.080]	0.391
Hcy T3	0.663 [−0.139, 1.464]	0.105	0.390 [−0.169, 0.949]	0.171	0.272 [−0.043, 0.588]	0.091	−3.274 [−6.165, −0.384]	0.027
Non-Optimal Sleep ^a^	0.142 [−0.796, 1.081]	0.766	0.195 [−0.460, 0.850]	0.558	−0.053 [−0.423, 0.317]	0.778	3.791 [0.406, 7.177]	0.028
Overwork ^b^	1.107 [0.455, 1.758]	0.001	0.729 [0.274, 1.183]	0.004	0.378 [0.121, 0.635]	0.004	−1.010 [−3.361, 1.341]	0.399
Non-Exercise Habit ^c^	0.974 [0.083, 1.865]	0.032	0.745 [0.124, 1.367]	0.019	0.229 [−0.122, 0.580]	0.201	−1.972 [−5.186, 1.242]	0.228
Dietary Variety Score	0.478 [−1.216, 2.172]	0.579	0.276 [−0.906, 1.458]	0.647	0.202 [−0.465, 0.870]	0.551	0.113 [−5.999, 6.225]	0.971

Hcy, homocysteine; ChaTF, Chalder total fatigue; ChaPF, Chalder physical fatigue; ChaMF, Chalder mental fatigue; VAS, visual analog scale. ^a^ ≥ 8 h, ^b^ < 5 h or ≥9 h. The model was adjusted with age, BMI, eGFR and ALT. ^c^ < 2 days in a week.

## Data Availability

The data presented in this study are available on request from the corresponding author due to privacy and ethical restrictions related to human participant data. Data may be available from the corresponding author upon reasonable request and with permission from the relevant ethics committee.

## Supplementary Materials

The following supporting information can be downloaded at: https://www.mdpi.com/article/10.3390/nu18060941/s1, Table S1: Comparison of baseline characteristics between participants included in the final analysis and those excluded due to missing biochemical or questionnaire data.
