# Supplementary material for: Associations of Plasma Homocysteine Reflecting Vitamin B12 and Folate Status with Fatigue-Related Outcomes in Healthy Adults
Source: Nutrients. 2026 Mar 17;18(6):941. doi: 10.3390/nu18060941 (PMC13028897; doi:10.3390/nu18060941)
Supplement: Supplementary file 1 [file nutrients-18-00941-s001.zip › nutrients-4194465-supplementary.pdf]

Supplementary Table S1. Comparison of baseline characteristics between participants included in the final analysis and those excluded due to missing biochemical or questionnaire data.

	**Men**			**Women**		
	**Excluded participants**	**Included participants**	***p***	**Excluded participants**	**Included participants**	***p***
	***n* = 578**	***n* = 204**		***n* = 1438**	***n* = 398**	
Age (years)	48(40−56)	42(34−51)	<0.0001	44(34−51)	45(34−52)	0.340
BMI (kg/m^2)	23.3(21.5−25.3)	22.4(21.0−24.4)	0.004	20.4(19.1−22.2)	20.6(19.1−22.5)	0.687
ALT (U/L)	21.0(16.0−29.0)	20.0(15.0−28.0)	0.390	13.0(10.0−18.0)	12(8−16)	0.002
eGFR (mL/min/1.73 m^2)	76.8(68.5−86.6)	80.0(71.6−91.1)	0.001	88.5(78.4−101)	83.1(74.2−95.2)	0.645
Creatinine (mg/dL)	0.86(0.79−0.94)	0.86(0.78−0.92)	0.216	0.62(0.56−0.68)	0.62(0.56−0.67)	0.333
d−ROMs (U.CARR)	331(288−375)	321(275−368)	0.272	397(341−460)	377(333−419)	<0.0001
BAP (µmol/L)	2332(2175−2471)	2415(2229−2539)	0.003	2346(2223−2471)	2373(2257−2504)	0.007
d−ROMs/BAP	0.14(0.12−0.16)	0.13(0.11−0.16)	0.026	0.17(0.14−0.20)	0.16 (0.14−0.18)	<0.0001
Non-Optimal Sleep	98(17)	28(14)	0.270	177(12)	39(10)	0.174
Overwork	342(59)	140(69)	0.015	337(23)	110(28)	0.079
Smoking Status Never	255(44)	84(41)		1179(82)	334(84)	
Former	222(39)	76(37)	0.357	188(13)	49(12)	0.573
Current	98(17)	44(22)		69(5)	15(4)	
Non-Habitual Exercise	470(81)	182(89)	0.086	1212(84)	351(88)	0.040
Dietary Variety Score	90.0(80.0−100)	85.7(71.4−100)	0.148	100(90.0−100)	100(75−100)	0.760
VAS Fatigue	28(17−48)	30(17−48)	0.664	34(21−52)	38(22−53)	0.263
VAS Sleepiness	31(16−49)	32(18.3−51.8)	0.314	35(19−53)	34(20−52)	0.886
VAS Depression	18(4−39)	17.5(0−35)	0.298	19(0−39)	24(9−43)	0.002
VAS Motivation	54(45−73)	60(48−76)	0.045	52(40−69)	51(41−67)	0.173
ChaTF	11(6−15)	10(6−14)	0.595	12(8−16)	12(8−16)	0.416
ChaPF	7(4−10)	6(4−9)	0.720	8(5-11)	7(5−11)	0.907
ChaMF	4(3−5)	4(2−5)	0.456	4(3−6)	4(3−6)	0.054

Categorical variables are presented as numbers and percentages, and continuous variables as medians with interquartile ranges (25th–75th percentile). Differences in continuous variables were assessed using Kruskal-Wallis test. Differences in categorical variables were assessed using Pearson's chi-square test.
